# A systematic literature review and network meta-analysis feasibility study to assess the comparative efficacy and comparative effectiveness of pneumococcal conjugate vaccines

**DOI:** 10.1080/21645515.2019.1612667

**Published:** 2019-06-19

**Authors:** Ashleigh McGirr, Shehzad M. Iqbal, Patricia Izurieta, Carla Talarico, Janneke Luijken, Josefine Redig, Rachel S. Newson

**Affiliations:** a GSK, Mississauga, Ontario, Canada; b GSK, Wavre, Belgium; c GSK, Rockville, MD, USA; d ICON plc, Houten, The Netherlands; e ICON plc, Stockholm, Sweden

**Keywords:** Pneumococcal conjugate vaccine, network meta-analysis, feasibility, invasive pneumococcal disease, comparative efficacy or effectiveness, systematic review

## Abstract

**Background**: No head-to-head studies are currently available comparing pneumococcal non-typeable *Haemophilus influenzae* protein D conjugate vaccine (PHiD-CV) with 13-valent pneumococcal conjugate vaccine (PCV-13). This study explored the feasibility of using network meta-analysis (NMA) to conduct an indirect comparison of the relative efficacy or effectiveness of the two vaccines.

**Methods**: A systematic literature search was conducted for published randomized controlled trials (RCTs) and non-RCT studies reporting data on vaccine efficacy or effectiveness against invasive pneumococcal disease in children aged <5 years receiving 7-valent pneumococcal conjugate vaccine (PCV-7), PHiD-CV or PCV-13. Study quality was evaluated using published scales. NMA feasibility was assessed by considering whether a connected network could be constructed by examining published studies for differences in study or patient characteristics that could act as potential treatment effect modifiers or confounding variables.

**Results**: A total of 26 publications were included; 2 RCTs (4 publications), 7 indirect cohort studies, and 14 case-control studies (15 publications). Study quality was generally good. The RCTs could not be connected in a network as there was no common comparator. The studies differed considerably in design, dose number, administration schedules, and subgroups analyzed. Reporting of exposure status and subject characteristics was inconsistent.

**Conclusion**: NMA to compare the relative efficacy or effectiveness of PHiD-CV and PCV-13 is not feasible on the current evidence base, due to the absence of a connected network across the two RCTs and major heterogeneity between studies. NMA may be possible in future if sufficient RCTs become available to construct a connected network.

## Introduction

Pneumococcal disease is caused by the bacterium *Streptococcus pneumoniae*. Over 90 serotypes of *S. pneumoniae* have been identified;^1^ however, the 10 most common serotypes account for approximately 62% of invasive pneumococcal disease (IPD) worldwide.^2^ The most common forms of IPD include pneumonia with empyema or bacteremia, meningitis and febrile bacteraemia.^3^ Non-invasive diseases such as middle ear infections (acute otitis media [AOM]), sinusitis and bronchitis are less severe and more common manifestations of pneumococcal infection.^3^ The World Health Organization (WHO) estimated in 2005 that pneumococcal disease was responsible for 1.6 million deaths annually worldwide, mainly concentrated in poorer countries.^3^ In the developed world, serious pneumococcal disease occurs mainly in children aged <2 years and in elderly people.^4^ IPD causes substantial mortality and morbidity, with 4,200 deaths and over 35,000 cases estimated in the USA in 2011.^2^

Routine immunization with pneumococcal conjugate vaccines (PCVs) has been shown to reduce hospitalization of children for pneumonia^5^ and has substantially reduced the incidence of IPD in Canada^6^ and other countries.^7^ The WHO recommends the inclusion of PCVs in childhood immunization programmes worldwide.^7^ In Canada, 7-valent PCV (PCV-7) was first licensed for use in 2001 and the National Advisory Committee on Immunization (NACI) recommended routine infant vaccination in 2002.^6^ NACI updated these recommendations to replace PCV-7 with pneumococcal non-typeable *Haemophilus influenzae* protein D conjugate vaccine (PHiD-CV, *Synflorix, GSK*) in 2009 and the 13-valent PCV (PCV-13, *Prevnar 13, Pfizer*) in 2010.^8^ PHiD-CV contains capsular polysaccharides of 10 pneumococcal serotypes: 1, 4, 5, 6B, 7F, 9V, 14, 18C, 19F, and 23F and also induces protection against cross-reactive serotype 19A.^9^ In Canada, PHiD-CV is licensed for immunization of infants and children from 6 weeks to 5 years of age,^10^ and PCV-13 is licensed for immunization of infants and children from 6 weeks to 17 years of age.^11^

There is a need for information on the comparative efficacy or effectiveness of PHiD-CV versus PCV-13 to help support decision-makers and health-care professionals selecting between available options. No head-to-head studies directly comparing PHiD-CV and PCV-13 are currently available.^12^ A recently published meta-analysis of randomized controlled trials (RCTs) on the efficacy of all PCVs showed a significant reduction in risk for IPD and no significant reduction in risk for death over placebo.^13^ This analysis did not attempt to compare between different PCVs. A recent systematic review of available evidence on the impact or effectiveness of PHiD-CV and PCV-13 in children aged <5 years in Latin American countries found that both PHiD-CV and PCV-13 were effective in reducing deaths or hospitalizations due to IPD, pneumonia, meningitis and sepsis, and there was no evidence of superiority of either vaccine over the other.^12^ However, meta-analysis was considered inappropriate because the review included studies with a wide variety of designs, endpoints and age stratification.

Network meta-analysis (NMA) is an established technique that allows for indirect comparisons between multiple interventions in the absence of direct head-to-head studies. As long as all the available trials have at least one intervention in common with another, a network can be constructed to link the interventions tested across the different trials. It is then possible to estimate relative effects for all interventions included in the network. This approach is commonly used to compare health-care interventions and has recently been applied to vaccines. Recommending and decision-making bodies have used NMAs to base their treatments guidelines on, such as the World Health Organization’s updated guidelines for treatment of Human Immunodeficiency Virus (HIV) and Hepatitis C Virus (HCV).^14^ More recently in vaccines, NACI commissioned an NMA in order to assess relative efficacy, effectiveness and safety of vaccines for herpes zoster,^15^ and an NMA has been used to evaluate the comparative effectiveness of two rotavirus vaccines.^16^ Given the complexity of the pediatric pneumococcal disease literature and the evolving landscape of new vaccine preparations, the use of NMA methodology to compare vaccines is a logical next step. The objective of this feasibility study was to assess whether NMA methodology could be used to evaluate the comparative efficacy or effectiveness of PHiD-CV and PCV-13 in preventing IPD in children aged <5 years, using RCTs and observational studies.

## Results

### Systematic literature review

**Figure 1.**
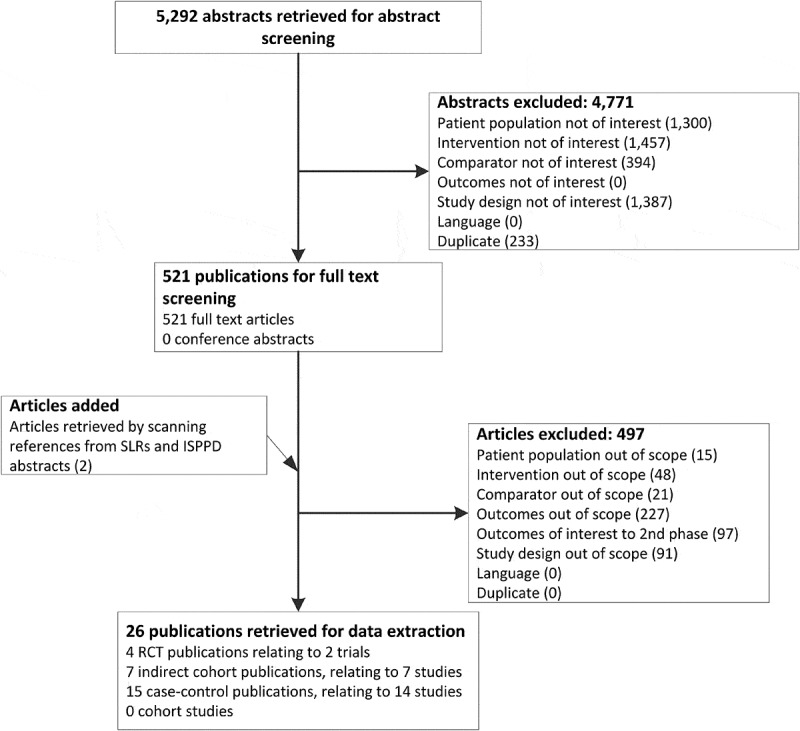
Flow diagram of search results and study selection. ISPPD, International Symposium on Pneumococci and Pneumococcal Diseases; RCT, randomised controlled trial; SLR, systematic literature review.

The literature search identified 5,292 publications, of which 521 were obtained for full-text review. Two additional publications were added from the International Symposium on Pneumococci and Pneumococcal Diseases (ISPPD) 2016 conference abstracts and other systematic literature reviews. After full-text review, 26 publications met the inclusion criteria and were included in the review (Figure 1). These consisted of 2 RCTs (4 publications),^17–20^ 7 indirect cohort studies^21-27^ and 14 case-control studies (15 publications).^28–42^

The key characteristics of the included studies are summarised in Table 1. Both RCTs were double-blind and multicentred. Of the non-RCTs, seven were indirect cohort analyses, all derived from national surveillance centers. In this type of study, the subjects with vaccine-type IPD (VT-IPD) act as the cases, while all other subjects with non-VT-IPD act as the controls. Fourteen studies were case-control studies, in which IPD/VT-IPD cases are compared to controls who do not have IPD/VT-IPD. All except two of these studies used matched controls, and the other two studies controlled for confounding factors. The duration of follow-up ranged from 2.5 to 9 years, and the studies were conducted in North America, South America, Europe, and Asia.

**Table 1.** Key study characteristics.

Author	Study design	Country	Arm name	Vaccine administered	N	Schedule	Dose
Black, 2000 Kaiser Permanente trial	RCT	US (Northern California)	PCV-7/intervention	Heptavalent pneumococcal conjugate, PCV-7	18,927	at 2, 4, 6 and 12 to 15 months of age	≥1 dose
			MCV/placebo	meningococcus type C conjugate vaccine	18,941	at 2, 4, 6 and 12 to 15 months of age	≥1 dose
Black, 2002 Kaiser Permanente trial	RCT	US (Northern California)	PCV-7/intervention	Heptavalent pneumococcal conjugate, PCV-7	18,927	at 2, 4, 6 and 12 to 15 months of age	≥1 dose
			MCV/placebo	meningococcus type C conjugate vaccine	18,941	at 2, 4, 6 and 12 to 15 months of age	≥1 dose
Shinefield, 2002 Kaiser Permanente trial	RCT	US (Northern California)	PCV-7/intervention	Heptavalent pneumococcal conjugate, PCV-7	18,927	at 2, 4, 6 and 12 to 15 months of age	≥1 dose
			MCV/placebo	meningococcus type C conjugate vaccine	18,941	at 2, 4, 6 and 12 to 15 months of age	≥1 dose
Tregnaghi, 2014 COMPAS, NCT00466947	RCT	Argentina, Panama, Colombia	PHiD-CV group	ten-valent pneumococcal non-typeable *Haemophilus influenzae* protein D conjugate vaccine (PHiD-CV)	23,821	3 + 1 PHiD-CV + DTPa-HBV-IPV/Hib at approx. 2, 4, 6 months and PHiD-CV + DTPa-IPV/Hib at 15–18 months	3 + 1
			Control group	Recombinant hepatitis B vaccine and hepatitis A vaccine	23,821	3 + 1 Hep B + DTPa-IPV/Hib at approx. 2, 4, 6 months and Hep A + DTPa-IPV/Hib at 15–18 months	3 + 1
Andrews, 2011	Indirect cohort	England and Wales	VT-IPD case	PCV-7	1,228	2-dose routine, at 2 and 4 months (evaluated for cases 5 months to <14 months); 2 dose older infants, at 3 and 8 months (evaluated for cases 5 months to <14 months); 2-dose routine + 1 (evaluated for cases ≥14 months); 2 dose older infants + 1 (evaluated for cases ≥14 months); 1-dose catch-up (evaluated for cases ≥14 months)	≥1 dose
			Non-VT-IPD control	PCV-7	1,228	2-dose routine, at 2 and 4 months (evaluated for cases 5 months to <14 months); 2-dose older infants, at 3 and 8 months (evaluated for cases 5 months to <14 months); 2-dose routine + 1 (evaluated for cases ≥14 months); 2-dose older infants + 1 (evaluated for cases ≥14 months); 1-dose catch-up (evaluated for cases ≥14 months)	≥1 dose
Andrews, 2014	Indirect cohort	England, Wales, Northern Ireland	VT-IPD case	PCV-13	716	2 + 1 schedule at 2, 4, and 12 months	≥1 dose
			Non-VT-IPD control	PCV-13	716	2 + 1 schedule at 2, 4, and 12 months	≥1 dose
De Serres, 2012	Indirect cohort	USA	VT-IPD case	PCV-7	1,447	according to age schedule	≥1 dose
			Non-VT-IPD control	PCV-7	1,233	according to age schedule	≥1 dose
Miller, 2011	Indirect cohort	England and Wales	VT-IPD case	PCV-13	264	according to age schedule, recommended schedule is 2 + 1 at 2, 4 and 13 months	≥ 1 dose
			Non-VT-IPD control	PCV-13	ND	according to age schedule, recommended schedule is 2 + 1 at 2, 4 and 13 months	≥ 1 dose
Ruckinger, 2010	Indirect cohort	Germany	VT-IPD case	PCV-7	30	according to age schedule, the recommended schedule is 3 + 1 at 2, 3, 4 and 11–14 months	≥1 dose
			Non-VT-IPD control	PCV-7	72	according to age schedule, the recommended schedule is 3 + 1 at 2, 3, 4 and 11–14 months	≥1 dose
van der Linden, 2016	Indirect cohort	Germany	VT-IPD case	PCV-7, PCV-13	617	according to age schedule, the recommended schedule is 3 + 1 at 2, 3, 4 and 11–14 months	≥1 dose
			Non-VT-IPD control	PCV-7, PCV-13	617	according to age schedule, the recommended schedule is 3 + 1 at 2, 3, 4 and 11–14 months	≥1 dose
Verani, 2015	Indirect cohort	Brazil	VT-IPD case	PHiD-CV	147	according to age, recommended PHiD-CV schedule includes 3 primary doses (2, 4, 6 months) + 1 booster (12 months). Catch-up schedules for children aged 3–11 months at the time of introduction included one to three primary doses (based on age) plus a booster dose; a single dose was recommended for children aged 12–23 months	≥1 dose
			VT-IPD case	PHiD-CV	75	according to age, recommended PHiD-CV schedule includes 3 primary doses (2, 4, 6 months) + 1 booster (12 months). Catch-up schedules for children aged 3–11 months at the time of introduction included one to three primary doses (based on age) plus a booster dose; a single dose was recommended for children aged 12–23 months	≥1 dose
			Non-VT-IPD control	PHiD-CV	94	according to age, recommended PHiD-CV schedule includes 3 primary doses (2, 4, 6 months) + 1 booster (12 months). Catch-up schedules for children aged 3–11 months at the time of introduction included one to three primary doses (based on age) plus a booster dose; a single dose was recommended for children aged 12–23 months	≥1 dose
Barricarte, 2007	Case-control	Spain	IPD case	PCV-7	85	3 PCV-7 doses, if the first dose occurred at 2–6 months of age; 2 doses, if the first dose occurred at 7–23 months of age; and 1 dose, if the first dose occurred at ≥24 months of age; a booster in the second year was also required for children who began vaccination before 12 months of age	≥1 dose
			Non-IPD control	PCV-7	425	3 PCV-7 doses, if the first dose occurred at 2–6 months of age; 2 doses, if the first dose occurred at 7–23 months of age; and 1 dose, if the first dose occurred at ≥24 months of age; a booster in the second year was also required for children who began vaccination before 12 months of age	≥1 dose
Ciruela, 2013	Case-control	Spain	IPD case	PCV-7	293	according to age schedule. PCV-7 has not been introduced into Spanish routine vaccination schedules (except for the community of Madrid in 2006), although the Spanish Paediatric Association recommends its use in children aged <2 years, with doses at 2, 4, 6 and 12–15 months. Schedule assumed to be 3 + 1 at months 2, 4, 6, and a booster at 12–15 months	≥1 dose
			Non-IPD control	PCV-7	782	according to age schedule. PCV-7 has not been introduced into Spanish routine vaccination schedules (except for the community of Madrid in 2006), although the Spanish Paediatric Association recommends its use in children aged <2 years, with doses at 2, 4, 6 and 12–15 months. Schedule assumed to be 3 + 1 at months 2, 4, 6, and a booster at 12–15 months	≥1 dose
Cohen, 2014	Case-control	South Africa	IPD case	PCV-7	237	2 + 1 schedule at 6 weeks, 14 weeks and 9 months	≥1 dose
			Non-IPD control	PCV-7	928	2 + 1 schedule at 6 weeks, 14 weeks and 9 months	≥1 dose
Deceuninck, 2010	Case-control	Canada	IPD case	PCV-7	298	Recommended schedule is 2 + 1 at 2, 4, and 12 months	≥1 dose
			Non-IPD control	PCV-7	4,549	Recommended schedule is 2 + 1 at 2, 4, and 12 months	≥1 dose
Deceuninck, 2015	Case-control	Canada	IPD case	PCV-7, PHiD-CV and PCV-13	889	Recommended schedule is 2 + 1 at 2, 4, and 12 months for PCV-7. For PHiD-CV and PCV-13, the recommended schedule is 2 + 1 at 2, 4 and 12 months	≥1 dose
			Non-IPD control	PCV-7, PHiD-CV and PCV-13	7,962	Recommended schedule is 2 + 1 at 2, 4, and 12 months for PCV-7. For PHiD-CV and PCV-13, the recommended schedule is 2 + 1 at 2, 4 and 12 months	≥1 dose
Domingues, 2014	Case-control	Brazil	IPD case	PHiD-CV	398	3 + 1, with three primary doses at 2, 4, and 6 months and a booster at 12 months	≥1 dose
			Non-IPD control	PHiD-CV	1,258	3 + 1, with three primary doses at 2, 4, and 6 months and a booster at 12 months	≥1 dose
Dominguez, 2011	Case-control	Spain	IPD case	PCV-7	293	Complete vaccination schedule is 3 doses at 2, 4, and 6 months and a fourth dose at 15 months, or 2 doses at least two months apart in children aged 12–23 months, or a single dose in children aged >24 months	≥1 dose
			Non-IPD control	PCV-7	751	Complete vaccination schedule is 3 doses at 2, 4, and 6 months and a fourth dose at 15 months, or 2 doses at least two months apart in children aged 12–23 months, or a single dose in children aged >24 months	≥1 dose
Fortunato, 2015	Case-control	Italy	IPD case	PCV-7 and PCV-13	39	3 PCV-13 doses at 3, 5–6, and 11–13 months of age. Children who had received one or two doses of PCV-7 completed their immunisation series with PCV-13. One PCV-13 catch-up dose was recommended for children fully vaccinated with PCV-7	≥3 doses
			Non-IPD control	PCV-7 and PCV-13	117	3 PCV-13 doses at 3, 5–6, and 11–13 months of age. Children who had received one or two doses of PCV-7 completed their immunisation series with PCV-13. One PCV-13 catch-up dose was recommended for children fully vaccinated with PCV-7	≥3 doses
Guevara, 2016	Case-control	Spain	IPD case	PCV-13	34	3 + 1, 3 doses at 2, 4, and 6 months and a booster dose at 12–15 months	≥1 dose
			Non-IPD control	PCV-13	272	3 + 1, 3 doses at 2, 4, and 6 months and a booster dose at 12–15 months	≥1 dose
Moore, 2016	Case-control	USA	IPD case	PCV-7, PCV-13	1,344	according to age schedule and the recommended schedule has 4 doses. PCV-7 was introduced in 2000, using a schedule of doses at 2, 4, 6 and 12–15 months of age. In 2010, PCV-13 replaced PCV-7 in the USA infant immunisation schedule	≥1 dose
			Non-IPD control	PCV-7, PCV-13	14,296	according to age schedule and the recommended schedule has 4 doses. PCV-7 was introduced in 2000, using a schedule of doses at 2, 4, 6 and 12–15 months of age. In 2010, PCV-13 replaced PCV-7 in the USA infant immunisation schedule	≥1 dose
Picon, 2013	Case-control	Uruguay	VT-IPD case	PCV-7	44	2 + 1 (2, 4, and 12 months) and a catch-up campaign with two doses (15 and 17 months)	≥1 dose
			Non-VT-IPD control	PCV-7	637	2 + 1 (2, 4, and 12 months) and a catch-up campaign with two doses (15 and 17 months)	≥1 dose
Pilishvili, 2010	Case-control	USA	IPD/VT-IPD case	PCV-7	1,267	≥1 dose	≥1 dose
			Non-IPD/non-VT-IPD control	PCV-7	8,018	≥1 dose	≥1 dose
Su, 2016	Case-control	Taiwan	IPD case	PCV-7, PHiD-CV and PCV-13	555	3 + 1 schedule, or one dose of catch-up schedule, or two doses of catch-up schedule, or 2 + 1 schedule	≥1 dose
			Non-IPD control	PCV-7, PHiD-CV and PCV-13	2,092	3 + 1 schedule, or one dose of catch-up schedule, or two doses of catch-up schedule, or 2 + 1 schedule	≥1 dose
von Mollendorf, 2015	Case-control	South Africa	IPD case	PCV-7	486	≥2 doses	≥2 doses
			Non-IPD control	PCV-7	2,037	≥2 doses	≥2 doses
Whitney, 2006	Case-control	USA	IPD case	PCV-7	1,267	≥1 dose, different schedules are possible but the recommended schedule is 3 + 1 at 2, 4, 6 and 12–15 months	≥1 dose
			Non-IPD control	PCV-7	8,018	≥1 dose, different schedules are possible	≥1 dose

All the included studies investigated the efficacy of a vaccine against IPD or VT-IPD. One RCT, the Kaiser Permanente trial, compared PCV-7 with the meningococcus type C conjugate vaccine (MCV).^17–19^ The other RCT, the COMPAS trial, compared PHiD-CV with the hepatitis B and hepatitis A vaccine.^20^ The non-RCTs all studied the effect of PCV-7, PHiD-CV, PCV-13 or combinations of these vaccines. PCV-7 was studied in 12 case-control studies and 4 indirect cohort studies, PHiD-CV was studied in 3 case-control studies and 1 indirect cohort study, and PCV-13 was studied in 5 case-control studies and 3 indirect cohort studies. All cases and controls in the non-RCTs received ≥1 dose of PCV in an age-appropriate schedule, but the schedules varied between studies. There was little information available on co-administered vaccines, dose volume and the route of administration, and no information on vaccine preparation or storage temperature.

### Feasibility of network meta-analysis for PCVs

#### Study quality assessment

**Figure 2.**
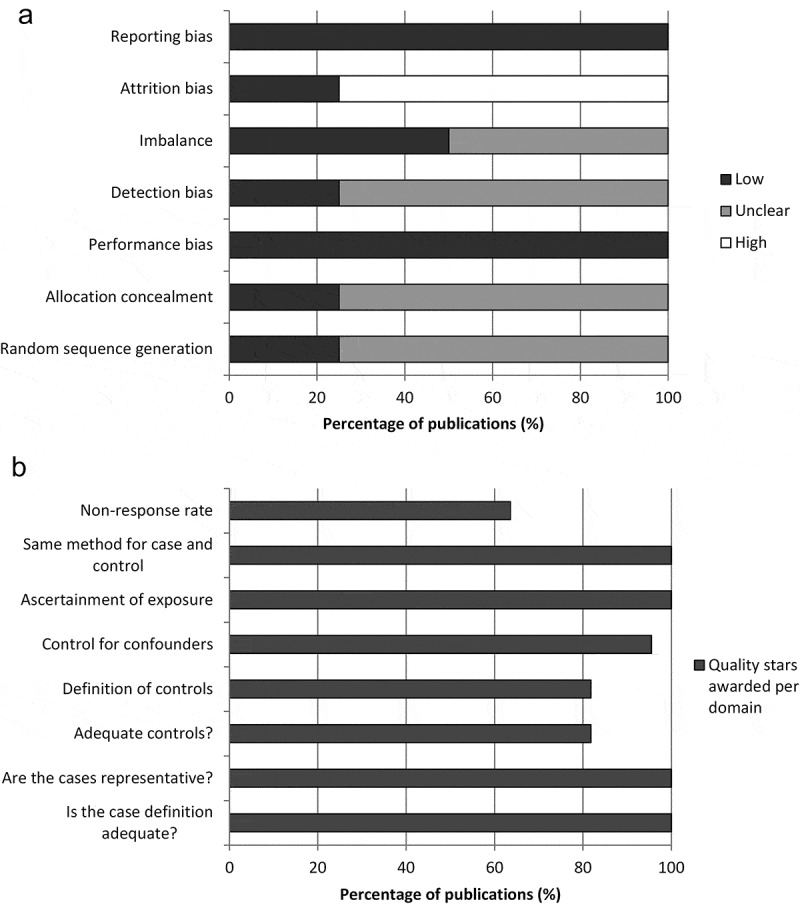
Summary of risk of bias assessment for (a) RCTs using the Cochrane risk of bias tool and (b) non-RCTs using the Newcastle-Ottawa scale. RCT, randomized controlled trial.

The risk of bias assessment for RCT and non-RCT studies is summarised in Figure 2.

The results of the risk of bias assessment for the two RCTs are shown in Supplemental Material 1. Of the two RCTs included in the review, the reporting of the Kaiser Permanente trial was unclear about the randomization process, the concealment of treatment allocation, blinding of outcome assessment and the number of subjects included or excluded. The reporting of the other RCT, the COMPAS trial, was considered to be clear according to the Cochrane Risk of Bias Tool, perhaps reflecting differences in reporting guidelines between the dates at which the two trials were conducted.

Of the 22 non-RCT publications, all had case definitions adequate for informing NMA. All also had representative cases as defined by the Newcastle-Ottawa tool (all eligible cases with outcome of interest over a defined period of time; all cases in a defined catchment area; all cases in a defined hospital, clinic, group of hospitals or health maintenance organisation; or an appropriate sample of cases [e.g. random sample]). Four publications used hospital controls instead of community controls, meaning that it would be challenging to combine the studies in NMA. Four did not have a good definition of controls. In seven publications the non-response rate was different between groups (e.g. the percentage participating was very different between cases and controls) or was not described.

#### Network structure

**Figure 3.**
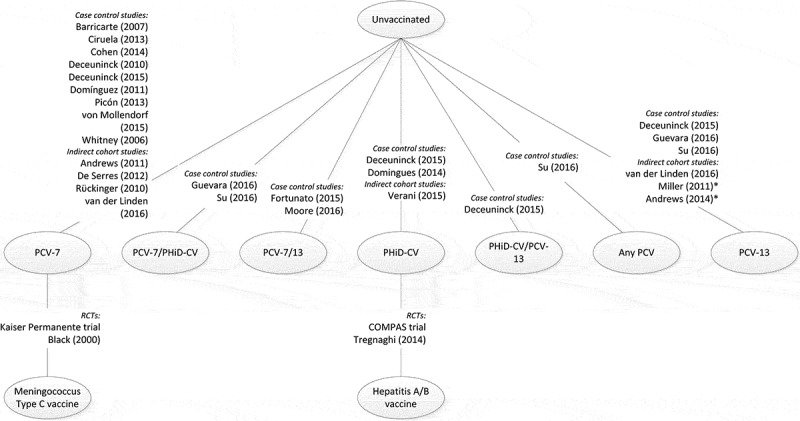
Overall network for included studies. PCV-7, 7-valent pneumococcal conjugate vaccine; PCV-13, 13-valent pneumococcal conjugate vaccine; PHiD-CV, pneumococcal non-typeable *Haemophilus influenzae* protein D conjugate vaccine; RCT, randomized controlled trial. *It cannot be assumed that exposure is solely PCV-13

The overall network structure for all the studies included in the review is shown in Figure 3. There was no linked network for the two RCTs, as one compared PHiD-CV with hepatitis A/hepatitis B vaccine, and the other compared PCV-7 with MCV, so there was no common comparator between the two studies (Figure 3).

The evidence base for conducting an NMA should first be based on RCTs. Non-RCT studies can be used to extend the network as a secondary analysis, however, using non-RCTs as the base for analysis is not robust. Thus, the network structure from the available publications did not allow for NMA, as there was no link across the network for the two RCTs (Figure 3).

#### Heterogeneity between studies

Heterogeneity between studies also precluded NMA. There was considerable heterogeneity in study designs, with three different types of studies identified (2 RCTs, 7 indirect cohort analyses and 14 case-control studies). In addition, the non-RCT studies also varied widely in location, date of publication, size, duration of follow-up and type of controls (17 studies used healthy community controls, and 4 studies used hospital controls). Combining evidence across heterogeneous designs has inherent difficulties due to differences in the measurement of vaccine efficacy or effectiveness. Indirect cohort studies and case-control studies have different types of controls (cases of non-VT-IPD, and controls without disease, respectively), and even within the group of case-control studies, different types of controls were used. There were also inconsistencies in the publications relating to one of the RCTs. These issues would introduce a high risk of bias if attempting to combine the studies in NMA.

There were large variations in dose number and administration schedules between studies (Table 1), with a different dose/schedule in almost every study. NMA relies on comparability of doses and schedules, so conducting an NMA using this heterogeneous evidence would only be possible if some of the disparate doses/schedules could be combined into fewer categories.

The number of vaccinated and unvaccinated subjects was not consistently reported across the included studies. Some studies reported only partial information on exposure (e.g. exposure information was missing for some outcomes or some study arms), while other studies did not report any information on exposure at all. Of the two RCTs, one reported complete exposure information together with the number of cases in each arm, while for the other RCT two of the three publications reported only the total number included across both arms. Of the seven indirect cohort studies, exposure was reported for both arms in five studies (however, two reported exposure data only for some outcomes), one study reported exposure only for one arm, and the other reported no exposure data at all. Of the 15 case-control publications, only seven reported full exposure data, two reported partial data on exposure (for one arm only), and the other six reported no exposure data. Overall, only about half of the included studies clearly reported the numbers of vaccinated and unvaccinated subjects for each analysis. It is essential to be certain about the numbers of vaccinated and unvaccinated subjects to conduct a valid NMA, and therefore the inconsistent exposure reporting makes the current evidence base unsuitable for NMA.

Outcome definitions for IPD and VT-IPD were highly comparable across studies.

There was substantial heterogeneity and little overlap across subgroups between the studies. Inconsistent categorization by age, dose/schedule, and serotypes between the different studies meant that over 750 different subgroups were analyzed or reported on across the studies, making it impractical to compare outcomes across the studies in an NMA. A robust NMA requires that similar subgroups are compared.

There was also heterogeneity in the risk of bias across the studies (Figure 2, Supplemental Material 1).

Patient characteristics should be similar across a network to minimize the risk of bias. The information reported on subject characteristics was limited, and inconsistent between the studies (Table 2). Age, sex and comorbidities were the most frequently reported, but even these parameters were not available in all studies. This lack of information on subject characteristics would make it difficult to assess or control for potential confounders in an NMA.

**Table 2.** Subject characteristics reported across studies.

Study	Age	Sex	Race^a^	CM	Premature birth	IC	Urban/rural
**RCT**							
Kaiser Permanente trial^17-19^					●		
COMPAS trial^20^	●	●	●	●			●
**Indirect cohort studies**							
Andrews 2011^21^		●	●	●	●		
Andrews 2014^22^							
De Serres 2012^23^		●		●			
Miller 2011^24^							
Ruckinger 2010^25^	●	●		●			
van der Linden 2016^26^		●					
Verani 2015^27^	●			●	●	●	
**Case-control studies**							
Barricarte 2007^28^		●					●
Ciruela 2013^29^		●		●		●	
Cohen 2014^30^	●	●		●	●		
Deceuninck 2010^31^			●	●	●		
Deceuninck 2015^32^							
Domingues 2014^33^	●	●		●	●	●	
Dominguez 2011^34^		●		●			
Fortunato 2015^35^		●					
Guevara 2016^36^	●	●		●	●		●
Moore 2016^37^	●	●	●	●	●	●	
Picon 2013^38^	●	●					
Su 2016^39^	●	●		●			
von Mollendorf 2015^40^				●			
Whitney 2006, Pilishvili 2010^41,42^	●	●	●	●	●	●	

^a^% caucasian CM, co-morbidities; IC, immunocompromised; RCT, randomised controlled trial

Overall, the results of the feasibility assessment indicated that it was not appropriate to use NMA to conduct an indirect comparison between PHiD-CV and PCV-13, due to the absence of a network connecting the RCTs and major heterogeneity between the available studies.

## Discussion

**Figure 4.**
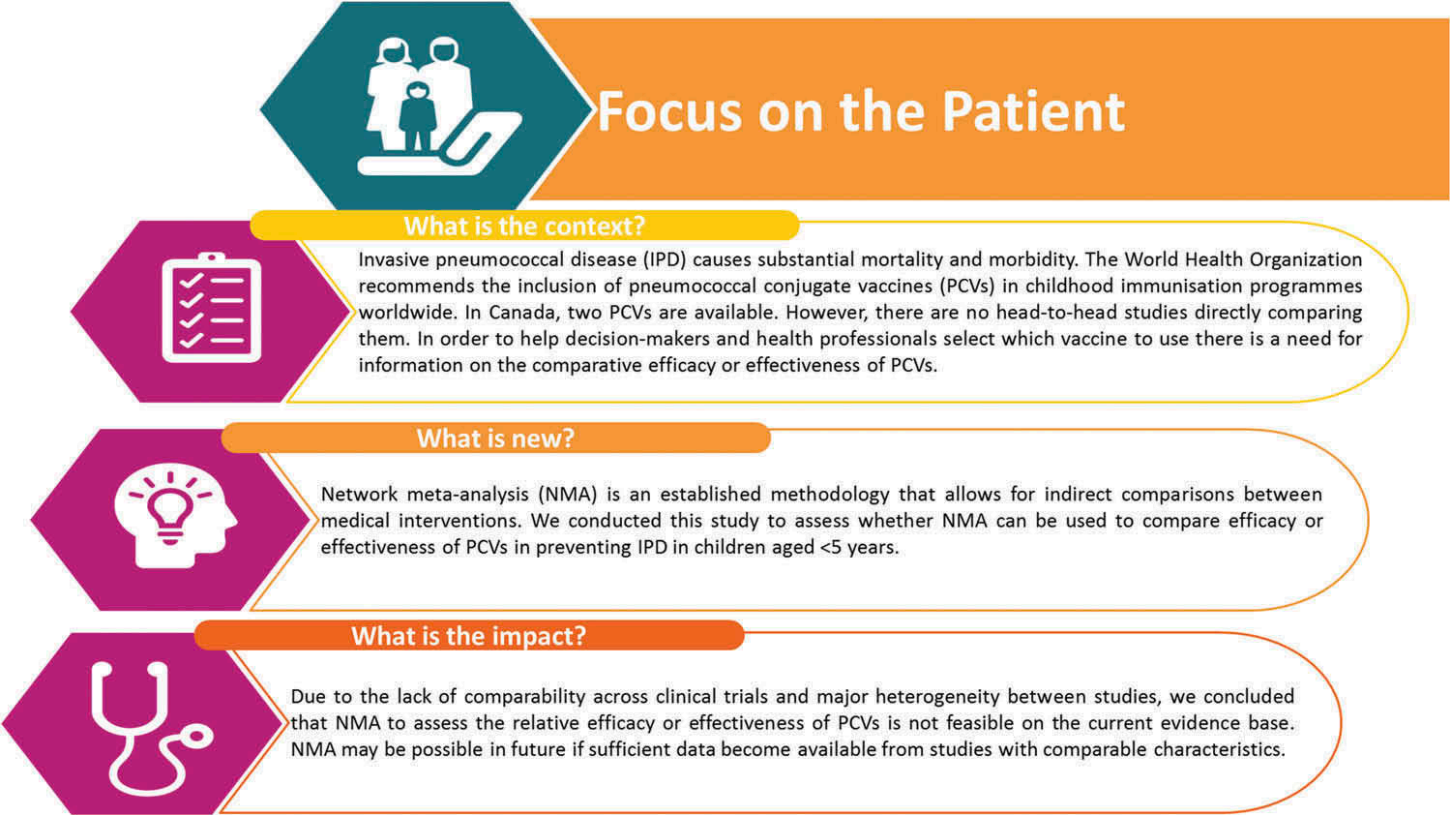
Summary of context, outcomes, and impact for health-care providers.

To our knowledge, this is the first attempt to explore the feasibility of using NMA techniques to investigate the relative efficacy or effectiveness of PHiD-CV compared with PCV-13. We identified 26 publications in a systematic literature review, relating to 2 RCTs, 7 indirect cohort studies and 14 case-control studies. These publications were assessed to determine the feasibility of conducting a NMA using them as an evidence base. Figure 4 presents a summary of the outcomes and the impact of this study for health-care providers. While there is a need for information on the comparative efficacy or effectiveness of PHiD-CV versus PCV-13, the current data do not support the use of NMA methodology.

The overall quality of the evidence base was good, with reporting quality assessed as relatively high for the non-RCTs, high for one RCT and low for the other RCT. However, the results of the feasibility analysis showed that an NMA would not be appropriate based on the publications identified. The first limiting factor is the absence of a network connecting the two RCTs. Although non-RCTs can be added to the network in a secondary step to strengthen the evidence base and improve generalisability, this is only relevant if there is a connected network of RCTs to build upon. The two RCTs included in the literature search were evaluating PHiD-CV and PCV-7, but there was no RCT found evaluating PCV-13. Licensure requirements set forth by the WHO did not require an RCT, but instead proof of non-inferiority by immunogenicity, demonstration of functional antibody response and induction of immunological memory were considered sufficient.^43^ As such, no RCT was required for the licensure of PCV-13 in the pediatric population, so the two RCTs in this analysis were for PCV-7 and PHiD-CV.

The second reason why NMA is not feasible is due to substantial heterogeneity across the studies in many dimensions. Outcome definitions for IPD and VT-IPD are comparable across studies. However, there are many differences in other dimensions such as study design (2 RCTs, 7 indirect cohort studies and 14 case-control studies), dose number, administration schedules and subgroups analyzed, and inconsistent reporting of exposure status and subject characteristics.

The risk of bias quality assessment indicated that the non-RCTs are of relatively high quality, so there would be little concern over using these studies in NMA. However, the two RCTs differ considerably in their risk of bias score, with the COMPAS study scoring highly and the reporting of the Kaiser Permanente study achieving only a low score. To conduct a robust NMA, the quality of the RCTs would need to be tested in scenario analysis, for which the evidence base is currently insufficient with only two RCTs.

As the different studies were conducted over a period of 16 years, it is not unusual that there would be substantial heterogeneity between them. There are other vaccine-preventable diseases for which the RCTs and other studies were conducted more similarly to one another, allowing for NMA to synthesize the comparable efficacy or effectiveness.^15,16^ While use of an NMA to evaluate the comparative efficacy or effectiveness of PCV-13 and PHiD-CV would allow for a relatively simple statistical analysis of existing data to address an important data gap, the current evidence base is insufficient to conduct and NMA. In the absence of head-to-head studies, public health decision-makers evaluating PCVs will need to rely on real-world evidence such as the reviews conducted by the WHO Strategic Advisory Group of Experts on Immunization^1^ and the Comité sur l’immunisation du Québec (CIQ),^44^ together with cost considerations.

Future research such as direct head-to-head studies or impact studies would be valuable to compare the two vaccines. For example, the Swedish health system allows the 21 counties to select either PCV-13 or PHiD-CV for their local immunization programmes, and approximately half the counties use each vaccine. Researchers in Sweden have recently used this unique opportunity to conduct an impact study comparing the two vaccines, which found no statistically significant difference in overall IPD incidence between counties using PCV-13 or PHiD-CV.^45^ This study is the nearest available to a head-to-head comparison; however, further studies are needed to corroborate the results.

If further RCTs using comparators common to the two existing RCTs are published in the future, a small NMA may become possible provided the studies are not too heterogeneous. Given that there are two pneumococcal conjugate vaccines currently in development,^46,47^ there may be RCTs evaluating these products in a pediatric setting that could be added to the network. However, it is unlikely that the addition of these potential studies would change the conclusion that an NMA is not feasible to evaluate the comparative efficacy/effectiveness of PHiD-CV and PCV-13. Emerging techniques, such as estimating effects based on similar comparators or the inclusion of single-arm studies,^48^ may also permit NMA if RCTs using different comparators or single-arm studies become available in the future. Should new-published evidence make a connected RCT base network possible in the future, adding evidence from non-RCTs could be considered to broaden the evidence from a small RCT network. If the heterogeneity is too great for this to be practical, the non-RCT evidence might be used to build information to conduct a Bayesian NMA.

## Conclusion

In conclusion, this feasibility study showed that NMA to compare the relative efficacy or effectiveness of PHiD-CV and PCV-13 is not appropriate using the current evidence base. The absence of a connected network across the two RCTs was a key limiting factor. However, even with a robust RCT base, the major heterogeneity between studies in design, dose, schedules, and subgroups analyzed, together with the inconsistent and limited reporting of subject characteristics and exposure status, makes synthesizing the current evidence impractical.

## Materials and methods

### Systematic literature review

The study investigated two research questions:

- What is the comparative efficacy of PCVs in preventing IPD in children?
- What is the comparative effectiveness of PCVs in preventing IPD in children?

A literature search was conducted on 17 May 2017 in OVID (including MEDLINE, MEDLINE in Process, EMBASE, and Cochrane Central Registry of Controlled Trials (CENTRAL)). Details of the search strategy used are provided in Supplemental Material 2. To ensure no studies were missed, the Cochrane Database of Systematic Reviews (CDSR) was also searched for existing systematic literature reviews on clinical efficacy of PCVs, and the abstracts from the 2016 ISPPD conference were manually searched, as this is the largest conference in the field of pneumococcal disease. The reference lists of two recent systematic literature reviews^1,12^ were also scanned.

Studies were selected for inclusion based on Population, Intervention, Comparators, Outcomes and Study design (PICOS) criteria as follows:

- **Population**: Healthy male and female children aged <5 years;
- **Interventions**: PCV-7, PHiD-CV, and PCV-13, regardless of the dose or schedule;
- **Comparators**: Any vaccine, placebo or unexposed cohort;
- **Outcomes**: IPD of all serotypes, and IPD of VT serotypes (VT-IPD);
- **Study design**: RCTs with vaccine efficacy data, and observational studies with vaccine effectiveness data (nested case-control studies and cohort studies were included if they reported individual comparative data). Vaccine impact studies were excluded.

Full details of the PICOS criteria are provided in Supplemental Material 3. The search was limited to human studies published from 1990 to the present. There were no language restrictions.

Publications identified by the search were initially screened against the PICOS criteria by two independent researchers using the title and abstract. For publications assessed as potentially relevant, full-text articles were obtained and evaluated against the same criteria. Any conflicts were resolved by a third independent researcher.

For each publication that met the selection criteria, data extraction was performed by one researcher and checked against the original study by an independent researcher. Data on study and patient characteristics were extracted to evaluate the comparability of the studies and patients. For outcomes, data on the incidence of IPD and/or VT-IPD were extracted from the text or tables in the publication where available. Data from figures were extracted using the Digitize-it software.^49^

RCTs included were assessed for internal (amount of selection, information and confounding bias) and external (generalisability of study results) validity, using the Cochrane Risk of Bias Tool, which has been tested for internal consistency, reliability and validity.^50^ Non-RCTs were assessed using the Newcastle-Ottawa tool for observational studies, which judges each study on three broad perspectives: the selection of the study groups; the comparability of the groups; and the ascertainment of either the exposure or outcome of interest.^51^

### Feasibility assessment for network meta-analysis

When considering the feasibility of using NMA as an appropriate evidence synthesis tool for a clinical field, the availability of data to create a network for each outcome, the quality of any included data, and any potential study heterogeneity need to be assessed. The feasibility of conducting a valid NMA was evaluated using a standardized approach.^52^ The first step was to assess whether there was a network of interlinked studies to allow comparisons between the vaccines, considering each study type and the combined studies for the overall network and for each outcome. The second step was to assess whether there were differences within or between direct treatment comparisons in study or patient characteristics that could act as potential treatment effect modifiers or confounding variables. Patient characteristics identified included age, sex, race, comorbidities, premature birth, immunocompromised status and area (urban versus rural). Study characteristics identified included study design, doses and schedules, number exposed (vaccinated versus unvaccinated), subgroups compared, outcome definitions and reporting, study quality, baseline risk, and relative vaccine efficacy. The results of the feasibility assessment determined whether it was feasible to conduct an NMA with the evidence available.

## Figures and Tables

**Table:** 

AOM	acute otitis media
CDSR	Cochrane Database of Systematic Reviews
CIQ	Comité sur l’immunisation du Québec
CM	co-morbidities
IC	immunocompromised
DTPa-HBV-IPV/Hib	diphtheria, tetanus, acellular pertussis, hepatitis B, inactivated poliomyelitis, and *Haemophilus influenzae* type b
IPD	invasive pneumococcal disease
ISPPD	International Symposium on Pneumococci and Pneumococcal Diseases
MCV	meningococcus type C conjugate vaccine
NACI	National Advisory Committee on Immunization
NMA	network meta-analysis
PCV	pneumococcal conjugate vaccine
PCV-7	7-valent pneumococcal conjugate vaccine
PCV-13	13-valent pneumococcal conjugate vaccine
PHiD-CV	pneumococcal non-typeable *Haemophilus influenzae* protein D conjugate vaccine
PICOS	Population, Intervention, Comparators, Outcomes, Study design
RCT	randomised controlled trial
SAGE	Strategic Advisory Group of Experts on Immunization
VT	vaccine-type
WHO	World Health Organization

## Trademark statement

*Synflorix* is a trademark of the GSK group of companies. *Prevnar* and *Prevnar 13* are trademarks of Pfizer Canada Inc.
