# Supplementary material for: A systematic literature review and network meta-analysis feasibility study to assess the comparative efficacy and comparative effectiveness of pneumococcal conjugate vaccines
Source: Hum Vaccin Immunother. 2019 Jun 19;15(11):2713–24. doi: 10.1080/21645515.2019.1612667 (PMC6930063; doi:10.1080/21645515.2019.1612667)
Supplement: Supplemental Material [file khvi-15-11-1612667-s001.docx]

A Systematic Literature Review and Network Meta-Analysis Feasibility Study to Assess the Comparative Efficacy and Comparative Effectiveness of Pneumococcal Conjugate Vaccines

Supplemental Material

Ashleigh McGirr^1^, Shehzad M. Iqbal^1^, Patricia Izurieta^2^, Carla Talarico^3^, Janneke Luijken^4^, Josefine Redig^5^, Rachel S. Newson^4^

^1^GSK, Mississauga, Ontario, Canada; ^2^GSK, Wavre, Belgium; ^3^GSK, Rockville, Maryland, USA; ^4^ICON plc, Houten, The Netherlands; ^5^ICON plc, Stockholm, Sweden

# Supplemental Material 1. Quality assessment of RCTs


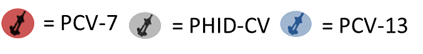


Green = Low Risk Yellow= Unclear Risk, Red = High Risk

PCV-13, 13-valent pneumococcal conjugate vaccine; PCV-7, 7-valent pneumococcal conjugate vaccine; PHiD-CV, pneumococcal non-typeable *Haemophilus influenzae* protein D conjugate vaccine; RCT, randomised controlled trial

# Supplemental Material 2. Search Strategy

Search strategy OVID

|  | **#** | **Search String** | **Hits** |
| --- | --- | --- | --- |
| Vaccine and Disease terms | 1 | Streptococcus pneumoniae/ | 60911 |
|  | 2 | streptococcus pneumoniae.tw. | 47515 |
|  | 3 | "s. pneumoniae".tw. | 19538 |
|  | 4 | exp Pneumococcal Infections/ | 31671 |
|  | 5 | (pneumococcal adj2 (infection* or disease*)).tw. | 12936 |
|  | 6 | (pneumococc* adj5 (pneumon* or sepsis or sinusit* or meningit* or otitis media)).tw. | 13455 |
|  | 7 | bacteraemic pneumon*.tw. | 124 |
|  | 8 | (invasive pneumococcal disease or ipd).tw. | 9103 |
|  | 9 | or/1-8 | 100454 |
|  | 10 | exp Vaccines/ | 507609 |
|  | 11 | exp Vaccination/ | 219274 |
|  | 12 | Immunization/ | 138176 |
|  | 13 | immunoprophylaxis.tw. | 5102 |
|  | 14 | (immuni* or inocul* or vaccin*).tw. | 1199909 |
|  | 15 | or/10-14 | 1371197 |
|  | 16 | 9 and 15 | 28892 |
|  | 17 | (pneumococcal conjugate or pneumococcus conjugate or conjugate pneumococcal or conjugate pneumococcus or pneumococcal polysaccharide conjugate or pneumococcal conjugated or pneumococcus conjugated or conjugated pneumococcal or conjugated pneumococcus or pneumococcal polysaccharide conjugated or pneumococcal conjugates or pneumococcus conjugates or conjugates pneumococcal or conjugates pneumococcus or pneumococcal polysaccharide conjugates or 7vcrm or 10vcrm or 11vcrm or 13vcrm or 9vcrm or 7vpnc or 10vpnc or 11vpnc or 13vpnc or 9vpnc or 7pcv or 10pcv or 11pcv or 13pcv or 9pcv or PCV-7 or PCV-13 or pcv11 or PHiD-CV or pcv9 or pcv 7 or pcv 10 or pcv 11 or pcv 13 or pcv 9 or pcv-7 or PHID-CV or pcv-11 or pcv-13 or pcv-9 or pncrm7 or pncrm10 or pncrm11 or pncrm13 or pncrm9 or pncrm-7 or pncrm-10 or pncrm-11 or pncrm-13 or pncrm-9 or seven-valent or ten-valent or eleven-valent or thirteen-valent or nine-valent or 7-valent or 10-valent or 11-valent or 13-valent or 9-valent or 7valent or 10valent or 11valent or 13valent or 9valent or 7 valent or 10 valent or 11 valent or 13 valent or 9 valent or PCV-7*13 or phid-cvi or phid10cv or phidcv or phidcv10 or phid-cv10 or heptavalent or synflorix or prevenar or prevenar 13 or prevnar or prevnar 13).mp. [mp=ti, ot, ab, sh, hw, kw, tn, dm, mf, dv, fx, nm, kf, px, rx, ui, sy] | 11884 |
|  | 18 | Pneumococcal Vaccines/ | 21852 |
|  | 19 | 16 or 17 or 18 | 38297 |
| RCT Filter | 20 | "randomized controlled trial".pt. | 883938 |
|  | 21 | (random$ or placebo$ or single blind$ or double blind$ or triple blind$).ti,ab. | 2933247 |
|  | 22 | (retraction of publication or retracted publication).pt. | 10747 |
|  | 23 | or/20-22 | 3122401 |
|  | 24 | controlled clinical trial.pt. | 183348 |
|  | 25 | randomized.ab. | 1229865 |
|  | 26 | drug therapy.fs. | 5367159 |
|  | 27 | randomly.ab. | 773196 |
|  | 28 | trial.ab. | 1192743 |
|  | 29 | groups.ab. | 4235871 |
|  | 30 | or/24-29 | 10458276 |
|  | 31 | (random$ or factorial$ or crossover$ or cross-over$ or placebo$ or (doubl$ adj blind$) or (singl$ adj blind$) or assign$ or allocat$ or volunteer$).mp. | 4376694 |
|  | 32 | crossover-procedure/ or double-blind procedure/ or randomized controlled trial/ or single-blind procedure/ | 969479 |
|  | 33 | or/31-32 | 4376694 |
|  | 34 | 23 or 30 or 33 | 12050748 |
|  | 35 | (animals not humans).sh. | 4367329 |
|  | 36 | exp animals/ not humans.sh. | 27329250 |
|  | 37 | ((comment or editorial or meta-analysis or practice-guideline or review or letter or journal correspondence) not "randomized controlled trial").pt. | 7626021 |
|  | 38 | (random sampl$ or random digit$ or random effect$ or random survey or random regression).ti,ab. not "randomized controlled trial".pt. | 153466 |
|  | 39 | or/35-38 | 31739690 |
|  | 40 | 34 not 39 | 4657988 |
|  | 41 | (random$ or placebo$ or single blind$ or double blind$ or triple blind$).ti,ab. | 2933247 |
|  | 42 | RETRACTED ARTICLE/ | 8402 |
|  | 43 | 41 or 42 | 2941425 |
|  | 44 | (animal$ not human$).sh,hw. | 8565758 |
|  | 45 | (book or conference paper or editorial or letter or review).pt. not exp randomized controlled trial/ | 8118761 |
|  | 46 | (random sampl$ or random digit$ or random effect$ or random survey or random regression).ti,ab. not exp randomized controlled trial/ | 151168 |
|  | 47 | or/44-46 | 16416540 |
|  | 48 | 43 not 47 | 2362150 |
|  | 49 | 40 or 48 | 5557596 |
| Non-RCT Filter | 50 | exp cohort studies/ | 2101529 |
|  | 51 | cohort$.tw. | 1092101 |
|  | 52 | controlled clinical trial.pt. | 183348 |
|  | 53 | Epidemiologic methods/ | 216677 |
|  | 54 | limit 53 to yr="1966-1989" | 110518 |
|  | 55 | exp case-control studies/ | 1010792 |
|  | 56 | (case$ and control$).tw. | 1015282 |
|  | 57 | or/50-52,54-56 | 3990892 |
|  | 58 | exp cohort analysis/ | 2101529 |
|  | 59 | exp longitudinal study/ | 328722 |
|  | 60 | exp prospective study/ | 910355 |
|  | 61 | exp follow up/ | 1153840 |
|  | 62 | cohort$.tw. | 1092101 |
|  | 63 | exp case control study/ | 1010792 |
|  | 64 | (case$ and control$).tw. | 1015282 |
|  | 65 | or/58-64 | 4981499 |
|  | 66 | 57 or 65 | 5230167 |
| Combining strings, adding limits, removing duplicates | 67 | 19 and 49 | 5219 |
|  | 68 | 19 and 66 | 5930 |
|  | 69 | limit 67 to human [Limit not valid in CCTR; records were retained] | 4536 |
|  | 70 | limit 69 to humans [Limit not valid in CCTR; records were retained] | 4536 |
|  | 71 | limit 68 to human [Limit not valid in CCTR; records were retained] | 5549 |
|  | 72 | limit 71 to humans [Limit not valid in CCTR; records were retained] | 5549 |
|  | 73 | limit 70 to english language | 4191 |
|  | 74 | limit 72 to english language | 5154 |
|  | 75 | remove duplicates from 73 ^a^ | 2774 |
|  | 76 | remove duplicates from 74 ^a^ | 3760 |
|  | 77 | 75 or 76 | 5877 |
|  | 78 | remove duplicates from 77 ^a^ | 5569 |
|  | 79 | limit 78 to yr="1990 -Current" | 5292 |
| **Total to Screen** | | | **5,292** |

^a^ de-duplicated with Embase as preference, followed by CCTR and Medline

# Supplemental Material 3. PICOS Criteria

| **Criteria** | **Stage** | **Inclusion** | **Exclusion** |
| --- | --- | --- | --- |
| Population | Abstract &  Full text selection | Children aged ≤5 years  Males and females | Studies where the majority of subjects are aged ≥5 years  Studies where the majority of subjects are immunocompromised |
| Intervention | Abstract & full-text selection | PCV-7, PHiD-CV, PCV-13 (all schedules and doses) | All other vaccines |
| Comparator | Abstract &  Full text selection | Any vaccine  Placebo  Unvaccinated individuals | No exclusions |
| Outcomes | Abstract selection | No selection on outcomes | No selection on outcomes |
|  | Full text selection | *Primary Outcomes*  Invasive pneumococcal disease of all serotypes (IPD)*;  Invasive pneumococcal disease of a pneumococcal serotype included in the vaccine administered (VT-IPD – including all serotypes included in the formulation of the vaccine plus the related serotype 19A)*;  *Secondary Outcomes*  All-Cause pneumonia*;  WHO defined pneumonia*;  Pneumococcal pneumonia*;  Otitis media/Acute Otitis Media*  VT Nasopharyngeal colonization* | Any outcomes not listed under inclusion |
| Study design | Abstract &  Full text selection | Randomized Controlled Trials  Observational effectiveness studies *(Including: (nested) case-control studies and cohort studies (study needs to report individual comparative data)* | Animal & cell studies;  Cost-effectiveness studies ;  Resource utilization studies;  Case reports;  Letters or replies to the editor or author;  Guidelines;  Single arm trials;  Cross-sectional studies  (Systematic) literature reviews**  Immunogenicity and bridging studies  Conference proceedings where no new data are reported compared to original trials  Pooled RCT analyses where no new data are reported compared to original trials  Vaccine impact studies |

* During screening definition ICD codes of IPD, pneumonia and (A)OM and whether PCR or lab culture confirmed were recorded.

** Reviews and meta-analyses were excluded from data extraction since pooled results cannot be used in analyses. However, good quality systematic reviews with effectiveness or efficacy data were used for cross-checking of references

IPD, invasive pneumococcal disease; PCV-7, 7-valent pneumococcal conjugate vaccine; PCV-13, 13-valent pneumococcal conjugate vaccine; PHiD-CV, pneumococcal non-typeable *Haemophilus influenzae* protein D conjugate vaccine; RCT, randomised controlled trial; VT, vaccine-type; WHO, World Health Organization
